# Drug-Induced Immune Thrombocytopenia Caused by Sulfamethoxazole-Trimethoprim: A Case Report

**DOI:** 10.5811/cpcem.65094

**Published:** 2026-10-30

**Authors:** Alec Powell, Louisa Weindruch, Jonathan Thornton

**Affiliations:** Baylor University Medical Center, Department of Emergency Medicine, Dallas, Texas

**Keywords:** drug-induced immune thrombocytopenia, thrombocytopenia, sulfamethoxazole-trimethoprim, mucosal bleeding, case report

## Abstract

**Introduction:**

Drug-induced immune thrombocytopenia (DITP) is a rare process in which specific drugs induce antibodies against platelet antigens, resulting in the destruction, consumption, or impaired production of platelets.^1^,^3^ Sulfamethoxazole-trimethoprim can result in DITP.^2^

**Case Report:**

This is a case of a 64-year-old man who developed severe thrombocytopenia after sulfamethoxazole-trimethoprim use. He presented to the emergency department with oral hemorrhagic lesions, mucositis, and petechiae. Laboratory evaluation revealed a low platelet count. Drug-induced immune thrombocytopenia was suspected, and the sulfamethoxazole-trimethoprim was discontinued. The patient’s platelet count responded rapidly after discontinuation of the offending drug and administration of dexamethasone.

**Conclusion:**

This case highlights the importance of early recognition of DITP and the avoidance of re-exposure to the causative agent. Prompt drug withdrawal and adjunctive corticosteroid therapy can facilitate complete clinical recovery.

## INTRODUCTION

Drug-induced immune thrombocytopenia (DITP) is caused by drug-specific antibodies that develop against platelet antigens, most commonly the glycoprotein IIb/IIIa complex (GPIIb/IIIa) and the glycoprotein Ib/IX complex (GPIb/IX),^1^ which can result in suppression of hematopoiesis, megakaryocytopoiesis inhibition, platelet destruction, and platelet consumption.^1^,^3^ This potentially life-threatening hematologic condition can be caused by drugs such as including quinine, carbamazepine, vancomycin, eptifibatide, sulfamethoxazole, and trimethoprim.^2^ We present a case of sulfamethoxazole-trimethoprim–induced thrombocytopenia presenting with oral mucosal bleeding, mucositis, petechiae, and a critically low platelet count in a patient who had a similar presentation with a prior exposure. This case highlights a rare complication of a commonly prescribed medication and demonstrates the importance of prompt recognition in the emergency department (ED) setting.

## CASE REPORT

A 64-year-old man with type 2 diabetes mellitus, hypertension, hyperlipidemia, and chronic urinary retention presented to the ED with several hemorrhagic oral lesions and a rash on his bilateral lower extremities. Four days prior to presentation, his chronic indwelling Foley catheter had been removed at an outside hospital. The patient, believing he had developed a urinary tract infection, started taking leftover sulfamethoxazole-trimethoprim belonging to his wife. Within three days, he developed hemorrhagic lesions on his tongue and a petechial rash involving his lower extremities. He reported dark stools two days prior to presentation but denied hematemesis, melena, and hematuria at presentation. He recalled a prior reaction to sulfa drugs that resulted in an episode of “low blood counts.”

The patient was well-appearing, in no acute distress, afebrile, and hemodynamically stable. On physical exam, he had hemorrhagic blisters on the tongue and lips (Image 1), mucositis, and petechiae involving the bilateral lower extremities (Image 2). No scleral icterus, lymphadenopathy, or hepatosplenomegaly was observed.

Laboratory evaluation was most notable for significant thrombocytopenia, with a platelet count of less than 1 × 10^3^ platelets/μL (reference range, 150–400 × 10^3^/μL). Hemoglobin and hematocrit were within normal limits.

Dermatology consult found the presentation not to be consistent with Stevens-Johnson syndrome or toxic epidermal necrolysis. The hematology team suspected drug-induced immune thrombocytopenia and recommended dexamethasone 40 mg intravenously (IV) in the ED, followed by daily doses while inpatient. Platelet transfusion and IV immunoglobulin were deferred due to absence of severe symptoms and active bleeding.

During his hospital stay, the patient’s platelet count and clinical findings improved rapidly after discontinuation of sulfamethoxazole-trimethoprim (Table). His hospital course was complicated by mild steroid-induced hyperglycemia, which was managed conservatively. The patient’s platelet count was adequately responding; therefore, hematology agreed without the decision to withhold the last dose of dexamethasone given the hyperglycemia. The patient was discharged on hospital day five after complete platelet recovery with instructions for repeat labs outpatient one week after discharge.

*CPC-EM Capsule*

What do we already know about this clinical entity?

*Drug-induced immune thrombocytopenia (DITP) is a rare process by which exposure to certain drugs results in production of antibodies against platelet antigens.*

What makes this presentation of disease reportable?

*We present a case of sulfamethoxazole-trimethoprim–induced thrombocytopenia presenting with oral mucosal bleeding and petechiae.*

What is the major learning point?

*Several medications can cause DITP and lead to significant thrombocytopenia. Early recognition, treatment, and avoidance of re-exposure is crucial.*

How might this improve emergency medicine practice?

*This case highlights a rare complication of a commonly prescribed medication and demonstrates the importance of prompt recognition in the emergency care setting.*

## DISCUSSION

Sulfamethoxazole–trimethoprim-induced immune thrombocytopenia is a well-known cause of DITP; use of other medications such as quinine, quinidine, vancomycin, penicillin, rifampin, ibuprofen, mirtazapine, oxaliplatin, suramin, carbamazepine, and ceftriaxone can cause this condition.^4^ A study by Kaufman et al reported 38 cases of DITP per million users of sulfamethoxazole-trimethoprim.^5^ It is most commonly caused by the production of immunoglobulin G antibodies that bind to platelet antigens, specifically GPIIb/IIIa and GPIb/IX.^1^

Thrombocytopenia is defined as a platelet count of less than 150 × 10^3^/μL^2^; however, in DITP, the platelet nadir is frequently less than 20 ×10^3^/μL.^1^ Onset typically occurs 5 to 10 days following drug exposure, resulting in the clinical manifestations of thrombocytopenia.^1^ Classic clinical signs include purpuric lesions on the skin and mucous membranes, hematuria, and gastrointestinal hemorrhage.^3^ Diagnostic confirmation is aided by platelet recovery after discontinuation and recurrence upon accidental re-exposure. In this patient, acute platelet reduction manifested shortly after initiating sulfamethoxazole-trimethoprim, consistent with a documented historical episode following exposure to the same agent.

When the specific causative agent cannot be definitively identified, discontinuation of all nonessential medications is warranted.^1^ As platelet count improves, the symptoms of thrombocytopenia will begin to subside, usually within 1 to 2 days (4 to 5 half-lives) after stopping the medication.^3^ Steroids can be used as an adjunct to increase the rate of platelet recovery. Intravenous immunoglobulin (IVIG) and platelet transfusions are typically reserved for severe bleeding, such as intracranial or intrapulmonary hemorrhage, or for persistent thrombocytopenia.^1^,^3^ Platelet transfusion may not show any benefit if the drug antibodies are still in the patient’s system.^1^

## CONCLUSION

This case demonstrates the clinical presentation of drug-induced immune thrombocytopenia and the recovery process, focusing on the rate of recovery and treatments that may accelerate it. It is important for clinicians to recognize drug-induced immune thrombocytopenia and consider new medications as possible causes. Immediate cessation of the offending agent is the primary therapeutic intervention for DITP. Steroids, IVIG, and platelets may serve as beneficial adjuncts depending on the clinical picture.

## Figures and Tables

**Image 1:**
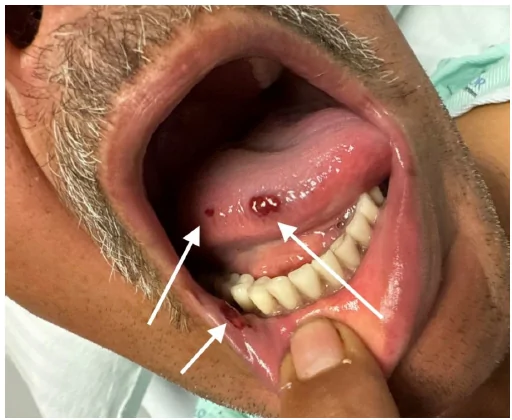
Hemorrhagic blisters seen on patient’s buccal mucosa and lateral tongue (arrows).

**Image 2:**
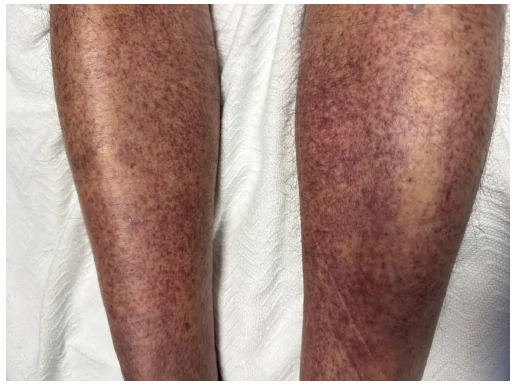
Extensive petechial rash on the patient’s lower extremities.

**Table:** Platelet count and hospital course.

Hospital Day	Platelet Count (×10^1^/μL)	Clinical Status
1	Less than 1	Oral mucosal hemorrhagic lesions, petechiae
2	11	Oral lesions improving
3	43	Petechiae resolving
4	85	Continued improvement
5	163	Complete recovery

*μL*, microliters.
